# Learning the Treatment Impact on Time-to-Event Outcomes: The Transcarotid Artery Revascularization Simulated Cohort

**DOI:** 10.3390/ijerph191912476

**Published:** 2022-09-30

**Authors:** Pablo Martínez-Camblor

**Affiliations:** 1 Biomedical Data Science Department, Geisel School of Medicine at Dartmouth, Hanover, NH 03755, USA; pablo.martinez-camblor@hitchcock.org; 2 Faculty of Health Sciences, Universidad Autonoma de Chile, Providencia 7500912, Chile

**Keywords:** Cox regression models, hazard ratios, marginal Cox regression models, time-to-event, survival analysis

## Abstract

Proportional hazard Cox regression models are overwhelmingly used for analyzing time-dependent outcomes. Despite their associated hazard ratio is a valuable index for the difference between populations, its strong dependency on the underlying assumptions makes it a source of misinterpretation. Recently, a number of works have dealt with the subtleties and limitations of this interpretation. Besides, a number of alternative indices and different Cox-type models have been proposed. In this work, we use synthetic data, motivated by a real-world problem, for showing the strengths and weaknesses of some of those methods in the analysis of time-dependent outcomes. We use the power of synthetic data for considering observable results but also utopian designs.

## 1. Introduction

A statistical model is a mathematical tool which tries to represent the data-generating process. As most of the mathematical tools, it bases on theoretical assumptions, which frequently suppose a simplification of the underlying reality. Crucial point is whether the model helps us to understand the studied phenomenon or it leads to misinterpretations.

Proportional hazard Cox regressions [1] are overwhelmingly used for modeling time-to-event outcomes. Their associated hazard ratios (HR) summarize the association between the variables of interest and the outcome along the follow-up period. Main advantage of these indices is, perhaps, their ability for including multivariate adjustments. However, these models have been the focus of a number of papers dealing with some of their limitations and common pitfalls. Cox regression models mainly assume that, if *T* is the random variable modeling the time-to-event of interest, then

(1) $$\begin{array}{cc} P(T>t|X=x,G=g)= & S(t|X=x,G=g)\\ = & \exp\{-\Lambda_{0}\left(t\right)\cdot \exp\left\{B_{X}\cdot x+B_{G}\cdot g\right\}\}, \end{array}$$

where S(·) and $\Lambda_{0}\left(\cdot \right)$ are the so-called survival and baseline cumulative risk functions, respectively, *X* is the studied variable (treatment), G a vector of covariates, and $B_{X}$ and $B_{G}$ are the logarithm of the hazard ratios associated with *X* and G, respectively.

Hernán [2] highlighted the relevance of the follow-up period in the HR estimation when the proportional hazard assumption is not hold. Stensrud and Hernán [3] insisted in the unrealistic assumption of the hazard proportionality in medical studies. Other authors, however, vindicate the hazard ratio as a useful measure for depicting the average differences between survival curves, proposing consistent estimators for the resulting parameter [4,5].

The non-collapsibility [6] of proportional hazard Cox regression models is, perhaps, more problematic. That is, since beyond the first observed event, the risk sets are comprised of the subset of individuals who has not previously failed, the immediate consequence is that the hazard ratio does not admit individual causal interpretation even in randomized clinical trials [7]. Or, in other words, if there exist factors affecting the time-to-event outcome do not included in the model, HRs cannot be identified even when those factors and the studied treatment were independently drawn.

A number of alternative Cox-type regression models have been proposed in order to avoid this issue. For instance, Lin and Ying [8] considered the additive model

$$\begin{array}{c} P\left(T>t|X=x,G=g\right)=\exp\{-\Lambda_{0}\left(t\right)+B_{X}\cdot x+B_{G}\cdot g\}. \end{array}$$

Similarly, additive-multiplicative models have been considered by Scheike and Shang [9]. MacKenzie et al. [10,11] considered a Cox-type model with the objective of, in the presence of omitted confounders, having an identifiable parameter. Wang et al. [12] and Martínez-Camblor et al. [13] considered an identifiable marginal structural Cox-type model. These models do not parameterize the potential effect of the unmeasured confounder and focus on the population-averaged hazard ratio. In this sense, the counterfactual is considered at populational level and the interpretation would be *what would have happened with the outcome if all the population would have been in the treatment/control group*.

Besides, there exist a number of alternative indices for summarizing the difference between survival curves. Royston and Parmar [14] considered the restricted mean survival time, a measure of the difference of survival time between the treated and control populations. Schemper et al. [5] considered several measures based on weighted average of the hazard ratio, which are interpreted beyond the proportional hazard Cox regression assumptions. Martínez-Camblor et al. [15] proposed a measured based on the area under the ROC curve which is the HR when the model is correctly specified and is easy to interpret in any case.

Data analysis is frequently a trade-off between what we actually want, and what the data allows us to know. In this work, we use synthetic data for analysing the effect of a treatment on a time-to-event outcome in presence of both measured and unmeasured confounding. The data generation was informed by a real-world problem. Particularly, we emulate a study in which the target was to compare the effectiveness of two procedures for treating carotid artery stenosis patients: transfemoral carotid artery stenting (TF-CAS), and a new procedure which use was approved in 2015, transcarotid artery revascularization (TCAR) [16]. The original study [17] included patients from the Vascular Quality Initiative (VQI) registry excluding patients who underwent carotid revascularization for reasons other than atherosclerotic disease or neointimal hyperplasia and those underwent carotid revascularization combined with another procedure.

This paper aims to show the strengths and weakness of time-to-event analyses. We focus on the limitations caused by the violations of the standard assumptions, specially, by the presence of unmeasured confounders. Rest of the document is organized as follows. In Section 2, we describe the real problem and explain the dataset generation process. Then, in Section 3, we study the behavior of the two populations (TF-CAS and TCAR) from different approaches: (i) unadjusted analyses, (ii) methods for considering the effects of measured confounding, (iii) methods for considering the effects of both measured and unmeasured confounding based on parametric assignment model, (iv) methods for dealing with both measured and unmeasured confounding considering marginal structural Cox models. In Section 4, we use additional information from the data-simulation process and considered non-reachable analyses. In one hand, we introduce the vector of unobserved confounders in both the conditional and the marginal Cox regression analyses, and in the other hand, we use the counterfactual generated times and report the results from a emulated randomized clinical trial. We discuss the results in Section 5. Finally, we provide some comments regarding the used software.

## 2. The Transcarotid Artery Revascularization Simulated Cohort

Carotid stenosis is treated with a combination of antiplatelet and cholesterol lowering medications, and procedural revascularization. There are three primary treatments for carotid revascularization: surgical carotid endarterectomy (CEA), transfemoral carotid artery stenting (TF-CAS), and a new procedure approved in 2015, transcarotid artery revascularization (TCAR). This new procedure has been rapidly adopted, however, TCAR was approved by the FDA without a randomized trial, and no comparative trial is underway. Therefore, observational studies are the only way to assess TCAR’s effectiveness and safety. Patients who receive TCAR are often different from patients who undergo CEA or TF-CAS in ways that are difficult to measure, including procedural selection effects, varying severity of comorbidity, and characteristics of the carotid lesion.

Among the studies comparing the effectiveness between TF-CAS and TCAR, recently, Columbo et al. [17] used data from the Vascular Quality initiative (VQI—www.vqi.org), which captures results on more than 95% of patients who undergo TCAR (https://silkroadmed.com/tcar-surveillance-project/) for studying perioperative and one-year rates of stroke or death. Here, we use the distributions obtained in that work for generating synthetic data and emulate the original problem in a controlled scenario. The data generation process was complex and included some manual changes. Final data were not generated from a single parametric model. This process helped us to emulate the distributions and numbers provided in the previous referred study. We simulated both time-to-event and censoring times and, appropriately, computed the observed times and the status at this point. We also simulated a measured covariate. This potentially plays the role of a score representing a linear combination of the usual measured confounders (including but not limited to Sex, Age, Race, Symptoms, Comorbidity, or ASA classification), and a unmeasured variable (it could include other unmeasured, or even unknown, factors affecting the outcome and, perhaps, also the treatment assignment, for instance, Genetic propensity or environmental risk exposures), both two variables were generated to be related with both the time-to-event and the treatment assignment. To note that those are not real, and they were not generated for representing, explicitly, any particular feature of the population but a general measured score. Finally, with the same process, we generated a variable just related with the treatment assignment which tries to reflect particular preference of the surgeons or centers for one particular treatment. All variables were computed with the objective of get the desired scenario. Final data are provided as online Supplementary Material.

## 3. Results

### 3.1. Unadjusted Analyses

**Figure 1:**
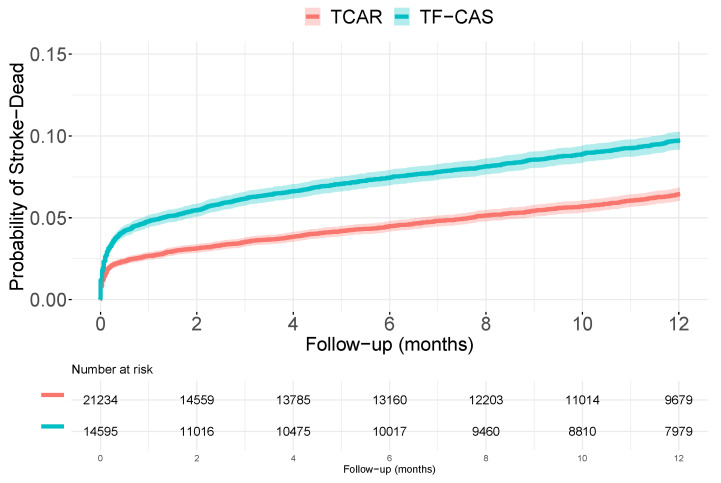
Kaplan–Meier estimations for the observed cumulative distribution functions of dying or having a stroke in both the TF-CAS and TCAR populations.

The dataset include a total of 35,829 patients, 14,595 underwent TF-CAS and 21,234 TCAR. During the follow-up (maximum of 12 months), 2168 patients suffered an stroke or died (had an event), 1166 and 1002 in the TF-CAS and TCAR groups, respectively. Within the first year after surgery, the overall percentage of having an event was 7.8 (95% confidence interval of [7.5 to 8.2]): 9.7 [9.2 to 10.2] vs. 6.4 [6.0 to 6.8] for the TF-CAS and TCAR patients, respectively. Figure 1 shows the Kaplan–Meier approximation for the cumulative distribution functions, CDF, of the time-to-event in both TF-CAS and TCAR groups.

Time-dependent outcomes use to be characterized through the so-called hazard function, λ(·). This function provides a dynamic description of the instantaneous risk of having the event at moment *t* being at risk until *t*. That is,

$$\lambda \left(t\right)=\lim_{\Delta \to 0}P\left(T<t+\Delta |T\geq t\right)/\Delta =-\partial \left\{\log\left[P\left(T>t\right)\right]\right\}/\partial t.$$

**Figure 2:**
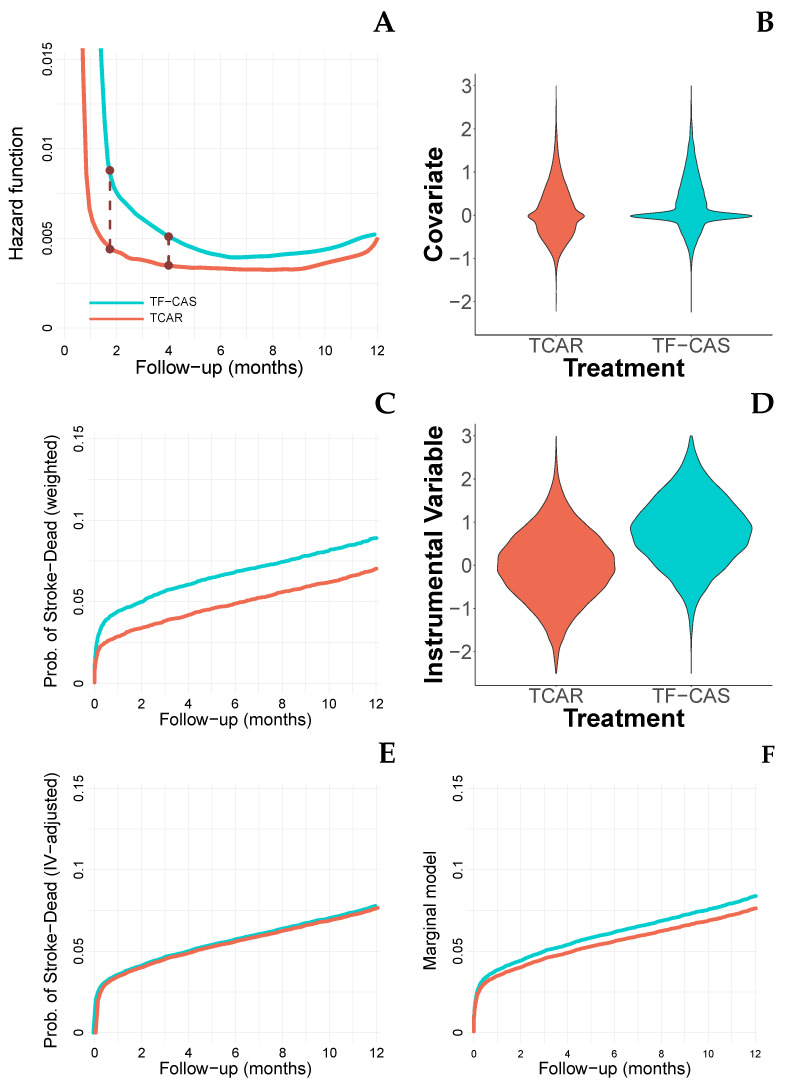
(**A**) Kernel-based estimation for the risk function in both TF-CAS and TCAR groups. (**B**) Violin-plots for the measured covariate by groups. (**C**) Propensity-score weighted Kaplan–Meier estimations by groups. (**D**) Violin-plots for the instrumental variable by groups. (**E**) Instrumental-variable adjusted survival curves. (**F**) Survival curves derived from the marginal model.

Figure 2A depicts the kernel-based estimation [18] for the hazard functions in both TF-CAS and TCAR groups. Proportional hazard Cox regression models assume that the quotient of these curves is constant. Since the estimation procedure of the associated HR (in this case, 1.57 with a 95% CI of [1.44 to 1.71]) gives more weight to those subjects with longer time at risk, the provided time-period HR is a weighted average of the actual time-dependent hazard ratio function. That is, let $\lambda_{0}\left(\cdot \right)$ and $\lambda_{1}\left(\cdot \right)$ be the hazard functions of having an event associated with the TF-CAS and the TCAR groups, respectively. Then, the time-period HR estimated through the standard partial likelihood involved in the Cox regression model is

$$\mathrm{wHR}=\int_{0}^{t_{f}}\frac{\lambda_{1}\left(t\right)}{\lambda_{0}\left(t\right)}dw\left(t\right),$$

where $\left[0,t_{f}\right]$ is the considered follow-up period and w(t) is a complex weighting function which depends, among other factors, on the distribution of the censoring time and the unmeasured covariates. Therefore, its interpretation beyond the proportional hazard model assumptions is not straight forward [19].

Notice that, in general, $\lambda_{x}\left(t\right)=E_{G_{x}}\left[\lambda_{x}\left(t|G_{x}=g\right)\right]$ (x∈{0,1}), where $G_{x}$ represents the (measured and unmeasured) features of the population involved in the problem. That is, the targeted $\lambda_{x}\left(\cdot \right)$ is the averaged risk of the subjects in the population x and, therefore, the observed behavior of this function depends on the distribution of those characteristic in the population. They are not exclusively affected by the treatment. Recall that the usual estimation process based on the optimization of the partial-likelihood function does not target an estimation of the average hazard ratio but a conditional hazard ratio for the semiparametric model reported in Equation (1).

Schemper et al. [5] considered to use different values of w(t). For w(t)=1, in our data, wHR was 1.54 [1.42 to 1.68]. Besides, the so-called concordance probability reported a value of 0.61 (highlight the coincidence between the odds, 0.61/0.39=1.56, and the hazard ratio reported by the regular conditional proportional hazard Cox regression estimation). That is, with a probability of 0.61, patients treated with TF-CAS are expected (at baseline) to die or having an stroke earlier than patients treated with TCAR.

Finally, the restricted mean survival time [14] directly considers the difference between the survival curves (RMST$\left(t_{f}\right)=\int_{0}^{t_{f}}\left[S_{0}\left(u\right)-S_{1}\left(u\right)\right]du$) avoiding the use of risk or hazard functions. Its value, −0.336 [−0.396 to −0.275], indicates that the average free of event time loss in the TF-CAS group during the first year after the procedure in comparison with the TCAR group was 0.336 months. Notice that, despite the other considered indices, RMST has units, which should be reported. Besides, the RMST is a measure referred to the population not to each particular subject.

### 3.2. Accounting for Measured Covariates

The role that the potential measured and unmeasured covariates plays in the observed difference between the studied groups is a major concern for establishing causal effects. When the covariates are measured, two main strategies use to be adopted. In the first considered one, we pursue that the initial characteristics of the studied groups have the same weight. Therefore, we will compare populations with similar (measured) features. In the second, we include the potential confounder in the model aiming to remove its effect on the observed results.

(a). We aim to compare populations with similar (measured) features. In this sense, we remove (or at least we reduce) the potential selection-bias. The propensity score-based procedures [20] are commonly used with this goal. First, for each subject, we compute its probability of being allocated in the treatment (non-treatment) group, and then, we balance these probabilities in both groups. With this goal, inverse propensity score weighting (IPSW) [21] and propensity score matching [22] are the most used techniques. Figure 2B shows the violin-plots for the measured covariate (here, it plays the role of a combination of the usual measured covariates including Sex, Age, Symptoms, Comorbidity, ASA classification, among others), Figure 2C shows the IPSW survival curves for both TF-CAS and TCAR populations [23]. The HR associated with the IPSW was 1.32 [1.21 to 1.44], very similar to the one provided by the matching procedure, 1.29 [1.17 to 1.142] (12,672 pairs satisfying the match). On this matched sample, the observed incidence percentages were 9.0 [8.6 to 9.6] vs. 7.6 [7.1 to 8.2] for the TF-CAS and TCAR patients, respectively. The incidence difference was, therefore, 1.4 [0.9 to 1.9]. The value of the weighted hazard ratio was 1.26 [1.15 to 1.39], and the value of the RMST was −0.21 [−0.28 to −0.15]. With these techniques, we pursue to fix the distribution of the measured confounders in both the TF-CAS and the TCAR groups, in this sense, we will be able to estimate parameters based on populations where the covariate distribution is the same in both considered groups. Notice that (i) those parameters can be affected by covariates in the same way that in the unweighted populations, (ii) the resulting population is mainly based on the intersection of the involved populations.

(b). When we aim to compare the patients in one group with their peers in the other group. That is, patients with a particular covariate value in the treatment group are compared with those patients with the same covariate value in the non-treatment group. Due the ambitious of this task, overall when we have continuous covariate, we have to make some assumptions. Particularly, we have to model the relationship between the outcome and the involved variables. Following the proportional hazard Cox regression model (Equation (1)), we have that the adjusted HR of the treatment conditioned to the measured covariate is 1.35 [1.24 to 1.47]. The well-known interpretation of this value is that, fixed the value of the covariate, the average hazard of having an immediate event of the patients in the TF-CAS group is 1.35 times more than the average hazard that similar patients have in the TCAR group. We evaluate the hazard functions $\lambda_{x}\left(t|M=m\right)=E_{G_{M}}\left[\lambda_{x}\left(t|M=m;G_{M}=g_{m}\right)\right]$ (x∈{0,1}), where $G_{M}$ includes all the variables related with the risk of event but *M*, and therefore a causal interpretation is still problematic. Besides, we assume that *M* impacts on the outcome linearly (Cox models allow some flexibility at this point) and that there is no interaction between the treatment and the covariate although the interaction terms could be also included in the model.

Doubly-robust inverse probability weighting [24], DR-IPSW, procedures combine both the inverse propensity score weighting (IPSW) and the inclusion of the the variables involved in the propensity score construction in the model. These procedures allow to compare patients on populations with similar characteristics and make comparisons only among those patients with similar characteristics, and they are more robust against erroneous specification of the outcome model when the propensity score model is correctly specified [25]. Here, the provided HR was 1.35 [1.24 to 1.47].

Measured covariate can also be considered in the weighted hazard ratio computation [5]. The direct adjusted HR average (w(t)=1) was 1.32 [1.21 to 1.44]. The concordance probability was 0.57 (odds of 0.53/0.47=1.13). Finally, the covariate-adjusted RMST was −0.221 months [−0.282 to −0.160].

### 3.3. Accounting for Unmeasured Covariates (I)

Despite the statistical tools available for dealing with the measured covariates, results derived from observational studies are always at risk of being influenced by the presence of unmeasured factors. Originally developed in economics [26], instrumental variable (IV) procedures help us dealing with unmeasured confounding. An IV is a variable which provides additional information about the treatment assignment mechanism, and that can be used in order to identify treatment effects. A valid IV, *the instrument*, should satisfy three key assumptions: (1) the instrument has to be related with the treatment assignment, (2) the instrument is independent of the outcome given the treatment, (3) the instrument is independent of unmeasured confounders. Besides, IV-based procedures usually require a fourth assumption related with the homogeneity of the treatment effects (further discussion about these assumptions can be found, for instance, in Lousdal [27]).

Although there is a number of instrument variable-based procedures, most of them do not consider Cox regression model particularities (non-collapsibility). Martínez-Camblor et al. [28] proposed the so-called two-stage residual inclusion-frailty (2SRI-F) algorithm, which reduces the bias of more standard two-stage procedures. In short, in the first-stage, 2SRI-F captures information related with the unmeasured confounder, and then incorporates it in the second-stage. A frailty term [29] helps to deal with the white-noise generated in the process.

So, in the first-stage, we save the residuals of the linear model,

Treatment=α·MeasuredCovariates+β·IV+γ.

Then, in the second-stage, we consider a frailty-Cox regression model which includes the treatment, the measured covariates, the residuals, and an individual parametric frailty term. To note that, in the the first-stage, the considered assignment model should always be linear, even when the studied treatment is binary. Despite binary treatments do not fulfil the required assumptions, and therefore, the theoretical properties are not guarantee, Monte Carlo simulations have shown [30] that 2SRI-F provides better results than its competitors. In this case, the 2SRI-F procedure reported a HR of 1.02 [0.83 to 1.24] (F-statistics associated with the IV variable was 8,383.23, which indicates a relatively strong instrument).

The average HR (w(t)=1) resulting of including the measured covariate and the residuals obtained from the first-stage in the 2SRI-F algorithm reports a value of 1.00 [0.81 to 1.22], with an associated concordance index of 0.5. The equivalent RMST was −0.027 months [−0.168 to 0.113].

Based on the 2SRI-F algorithm, Martínez-Camblor et al. [31] provided a semi-parametric procedure for adjusting survival curves for measured and unmeasured covariates. These curves can also be directly used for estimating the RMST which, in this case, provides a value of −0.012 months. Figure 2E shows the IV-adjusted survival curves for the TF-CAS and TCAR patients.

### 3.4. Accounting for Unmeasured Covariates (II)

Standard two-stage instrumental variable procedures require of strong parametric and non-parametric assumptions. Besides, the non-collapsibility of the Cox regression models makes that the derived hazard ratios are not identifiable when an unmeasured covariate, independently drawn, is affecting the time-to-event outcome. In this case, since the unmeasured covariate is not involved in the treatment selection process, the IV gets no information about this covariate from the assignment model. Wang et al. [12] and Martínez-Camblor et al. [13] proposed different estimators for the so-called marginal structural Cox regression model. Basically, this model assumes that not the conditional but the marginal risk function follows a Cox-type model, that is, it assumes the equality

$$\lambda_{1}^{T}\left(t\right)=\lambda_{0}^{T}\left(t\right)\cdot e^{\psi },$$

where $\lambda_{d}^{T}\left(\cdot \right)$ (d∈{0,1}) stands for the potential risk function if the unit were exposed to *d*, with independence of which the actual exposure was. In practice, *the unit* is the population of reference and the underlying model is

$$\begin{array}{cc} E_{G}\left[S_{1}\left(t;G=g\right)\right]= & \left\{\int S_{1}\left(t;g\right)dF_{G}\left(g\right)\right\}\\ = & {\left\{\int S_{0}\left(t;g\right)dF_{G}\left(g\right)\right\}}^{e^{\psi }}=E_{G}{\left[S_{0}\left(t;G=g\right)\right]}^{e^{\psi }}. \end{array}$$

This model is not affected by independent unmeasured confounders and, therefore, it is identifiable from a randomized clinical trial. The so-called causal hazard ratio [12], $e^{\psi }$, is at populational level. That is, the counterfacual would be *which would have been the behavior of the population if all (none) subjects would have had the treatment*.

The reported unadjusted estimations for the Martínez-Camblor et al. [13] and Wang et al. [12] procedures were 1.09 [0.86 to 1.39] and 1.08 [0.88 to 1.32], respectively. Furthermore, when we consider weighting by the measured confounder, they were 1.09 [0.90 to 1.33] and 0.93 [0.75 to 1.14], respectively. Figure 2F shows the curves reported by the weighting procedure proposed in Martínez-Camblor et al. [13].

## 4. Non-Reachable Analyses

### 4.1. Knowing the Unmeasured Covariate

**Figure 3:**
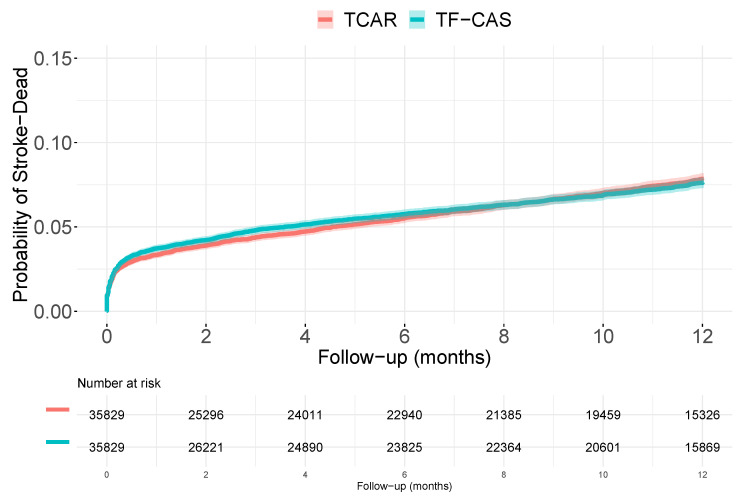
Kaplan–Meier estimations for the cumulative distribution functions of dying or having a stroke when the whole population undertake TF-CAS / TCAR.

The advantage of synthetic data is that we know the reality underlying the data generation process. In this case, we have access to the real *unmeasured* variable values. The proportional hazard Cox regression model, including the measured and *unmeasured* covariates, reported a HR of 1.03 [0.93 to 1.13]. The weighted HR (w(t)=1) was 1.62 [1.50 to 1.76], with a concordance probability of 0.61, while the RMST adjusted by both measured and unmeasured covariates was 0.004 months [0.003 to 0.005]. Besides, using the same generation process, with each observed survival time, we generated its counterfactural, that is, the time which would have been observed if the patient would have received the opposite procedure to the one he/she actually received. Figure 3 shows the probability of having an event when all patients undertake TF-CAS and when all patients undertake TCAR.

### 4.2. A Randomized Clinical Trial

**Figure 4:**
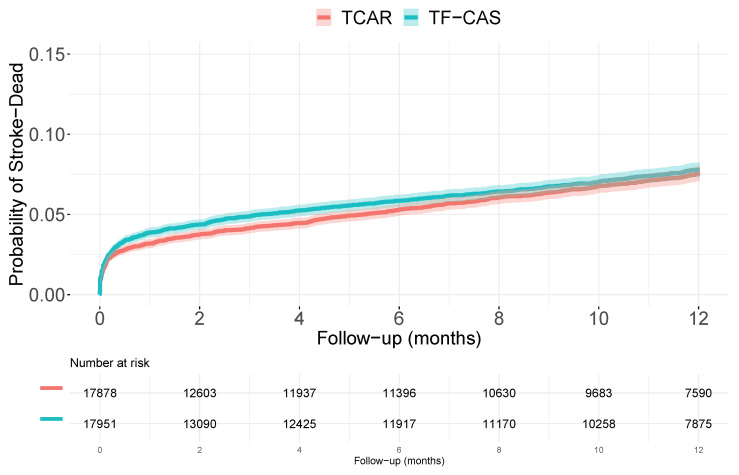
Kaplan–Meier estimations for the cumulative distribution functions of dying or having a stroke in the simulated RCT.

Finally, we emulate a randomized clinical trial experiment. Each one of the 35,829 patients is randomly assigned to the TF-CAS or to the TCAR treatment (with probability 1/2). A total of 17,951 patients were randomly assigned to receive TF-CAS while 17,878 received TCAR. Kaplan–Meier estimator reported percentages of event of 7.8 [7.3 to 8.2] and 7.5 [7.1 to 8.0] in the TF-CAS and TCAR groups, respectively. The hazard ratio resulting from the regular proportional hazard Cox regression model was 1.05 [0.97 to 1.15], and in this case, this would be also the hazard ratio associated with the marginal structural Cox model. The weighted hazard ratio (w(t)=1) was 1.02 [0.93 to 1.12] and the RMST was −0.061 months [−0.12 to −0.004]. Figure 4 shows the Kaplan–Meier estimation for the cumulative distribution function for the TF-CAS and TCAR groups.

## 5. Discussion

Selection bias is a major issue in the interpretation of the results derived from observational designs. Besides, if we consider a non-linear model, the non-collapsibility can also be a relevant issue. In the considered problem, direct unadjusted analyses of the time-to-event outcome report small but consistent difference between the TF-CAS and TCAR groups. TF-CAS had 1.4% more percentage of events, during the follow-up, the risk of having an event in TF-CAS was 50% greater than in the TCAR group. The time lost in the TF-CAS group was around 0.3 months. When we consider the measured covariate, the difference between groups dilutes a little bit. The used indices report hazard ratios around 1.3 and the time lost for those patients in the TF-CAS group if the measured covariate had were the same would be 0.2 months. When we try to get some information of the unmeasured confounder, and incorporate this information in the models, the difference mainly disappear, and the confidence intervals included the *no effect* value. Curiously, when we introduce the real unmeasured variable, the weighted hazard ratio returns to the beginning, with a wHR of 1.6. However, rest of the parameters are also very close to their *no effect* value. Discrepancies between HR and wHR were probably caused by misspecifications in the Cox model (data were not generated from this model). The simulated randomized clinical trail also reported no difference between the groups. Both potential distribution functions (Figure 3), and those obtained in the RCT (Figure 4) were very similar and reflect the (actual) no-effects situation. Of course, data were generated under *the no-effect* scenario. Table 1 summarizes most of the numbers (effect sizes measures and their respective 95% confidence intervals) reported along the paper.

Conventional causal inference concerns about an unobservable and relatively unuseful quantity: *what would have happened to one patient if their treatment would have been different to the one it actually was*. The estimation of this quantity is especially complex when the outcome of interest is a time-dependent variable. In biomedicine, the interest of this quantity would be to know what is the best treatment to use in future patients, that is, what we can learn is which would be the effect in patients like those on which we did the estimation. Therefore, strictly speaking, we are interested in populations. In this sense, hazard ratios provided by the conditional proportional hazard Cox regression models derived from adequate design can have causal interpretation, although, for doing that, we have to consider the marginal structural Cox regression model and to consider a population-averaged causal interpretation.

We also wanted to highlight here the difference between using adjusted and weighted models. Perhaps, the usual language used for referring to those procedures does not help in highlighting the difference between them. However, we would like to remark the different philosophies behind both approaches.

In this work, we have shown that the presence of unmeasured confounder can produce considerable bias in the observed results. In the statistical literature, there exist a number of tools, which helps to summarize and interpret time-to-event outcomes. All of them have different properties, which help to understand what the underlying data generation process could have been. We also have some procedures for dealing with unmeasured covariate although those frequently require strong assumptions and/or particular models. In practice, one never knows how the unmeasured factors can alter the observed reality and some caution in the interpretation is always welcome. Usually, more raw measures, such as the incidence differences or the restricted mean survival time, require less assumptions, and therefore, they are more robust. Unfortunately, additional conditions have to be introduced in order to deal with measured and unmeasured confounding. Hazard ratios derived from proportional hazard ratio Cox regression models are popular and easy to interpret. They can directly accommodate potential confounders, and other alternatives such as interactions or time-varying outcomes. The subtleties around its interpretation when the underlying model assumptions are violated is, without doubts, its major handicap.

We do not think that statistical science tolerates universal recipes. However, we include some tips for dealing with time-to-event-outcomes:

- Report raw survival curves and raw measures such as incidence difference and/or RMST, including confidence intervals.
- Report the HRs for the unadjusted model, also the results including different covariates. Consider the use of propensity score weighting and/or matched samples.
- In general, consider the HR as a measure of the difference between the distributions, not with a close interpretation which strongly depends on the underlying assumptions.
- [Just in case] Consider different IV procedures, including marginal models, and interpret the results with caution.

Perhaps, the main conclusion of this document is (again) that we *should thus focus on describing accurately how the study was conducted, what problems occurred, what data were obtained, what analysis methods were used and why, and what output those methods produced* [32].

## 6. Computational Details

Fortunately, nowadays, there exist a wide variety of resources that allow to implement most of the statistical analyses. Here, we have use the environment R (www.r-project.org). Particularly, we use the package survival [33], developed by Therneau et al., which includes a number of procedures for dealing with time-dependent outcomes including regular proportional hazard Cox regression models with and without frailty terms. The weighted hazard ratio and the restricted mean survival time difference were computed with the package coxphw [34] and survRM2 [35], respectively. The functions for computing the estimation of the marginal structural Cox regression model proposed by Martínez-Camblor et al. [13] were provided as online Supplementary Material in that paper. For the estimator proposed by Wang et al. [12], we used the implementation provided in the manuscript. The package muhaz [36] was used for estimating the hazard functions provided in Figure 2.

## Figures and Tables

**Table 1:** Summary. Punctual and 95% confidence intervals for some of measured reported along the paper: incidence difference (ID), conditional hazard ratio (HR), weighted hazard ratios (wHR), restricted mean survival times at twelve months (RMST), and marginal hazard ratios (mHR) in the different considered situations: unadjusted models (Crude), match-sample (Measured (a)), models adjusted by measured confounders (Measured (b)), models adjusting by both measured and no measured confounders (Omitted), and results provided by the RCT.

	Crude	Measured (a)	Measured (b)	Omitted	RCT
**ID**	3.3 [2.7 to 3.9]	1.4 [0.9 to 1.9]			0.3 [−0.2 to 1.2]
**HR**	1.57 [1.44 to 1.71]	1.29 [1.17 to 1.42]	1.35 [1.24 to 1.47]	1.02 [0.83 to 1.24]	1.05 [0.93 to 1.12]
**wHR**	1.54 [1.42 to 1.68]	1.26 [1.15 to 1.39]	1.32 [1.21 to 1.44]	1.00 [0.81 to 1.25]	1.02 [0.93 to 1.12]
**RMST**	−0.3 [−0.4 to −0.3]	−0.2 [−0.3 to −0.1]	−0.2 [−0.3 to −0.2]	−0.03 [−0.2 to 0.1]	−0.1 [−0.1 to −0.01]
**mHR**	1.09 [0.86 to 1.39]	1.09 [0.90 to 1.33]			1.05 [0.97 to 1.15]

## Data Availability

The document is based on synthetic data. Data are provided as supplementary material.

## Supplementary Materials

The following supporting information can be downloaded at: https://www.mdpi.com/article/10.3390/ijerph1010000/s1.
